# Sequencing of *NOTCH1*, *GATA5*, *TGFBR1* and *TGFBR2* genes in familial cases of bicuspid aortic valve

**DOI:** 10.1186/1471-2350-14-44

**Published:** 2013-04-11

**Authors:** Ilenia Foffa, Lamia Ait Alì, Paola Panesi, Massimiliano Mariani, Pierluigi Festa, Nicoletta Botto, Cecilia Vecoli, Maria Grazia Andreassi

**Affiliations:** 1 CNR Istituto di Fisiologia Clinica, Via Moruzzi 1, Pisa, 56124, Italy; 2 Fondazione CNR-Regione Toscana Gabriele Monasterio, Pisa, Italy

**Keywords:** Bicuspid aortic valve, Direct gene sequencing, Genes

## Abstract

**Background:**

The purpose of our study was to investigate the potential contribution of germline mutations in *NOTCH1*, *GATA5* and *TGFBR1* and *TGFBR2* genes in a cohort of Italian patients with familial Bicuspid Aortic Valve (BAV).

**Methods:**

All the coding exons including adjacent intronic as well as 5^′^ and 3^′^ untranslated (UTR) sequences of *NOTCH1*, *GATA5*, *TGFBR1* and *TGFBR2* genes were screened by direct gene sequencing in 11 index patients (8 males; age = 42 ± 19 years) with familial BAV defined as two or more affected members.

**Results:**

Two novel mutations, a missense and a nonsense mutation (Exon 5, p.P284L; Exon 26, p.Y1619X), were found in the *NOTCH1* gene in two unrelated families. The mutations segregated with the disease in these families, and they were not found on 200 unrelated chromosomes from ethnically matched controls. No pathogenetic mutation was identified in *GATA5*, *TGFBR1* and *TGFBR2* genes.

**Conclusions:**

Two novel *NOTCH1* mutations were identified in two Italian families with BAV, highlighting the role of a *NOTCH1* signaling pathway in BAV and its aortic complications. These findings are of relevance for genetic counseling and clinical care of families presenting with BAV. Future studies are needed in order to unravel the still largely unknown genetics of BAV.

## Background

Bicuspid aortic valve (BAV) is the most common congenital cardiac malformation, with a prevalence estimated between 0.5% and 2% [1,2]. Nearly all patients with BAV develop serious aortic complications, including significant valve regurgitation, severe aortic stenosis, aortic aneurysm and dissection [3,4]. Therefore, the burden of BAV disease is more significant than any other congenital cardiac abnormalities [4]. Clinical studies have reported high heritability in families of patients with BAV [5-7], suggesting a Mendelian pattern of inheritance and the need for echocardiography screening in first-degree relatives of patients with BAV [8].

A family-based linkage analysis has identified genomic regions on chromosomes 18q, 5q and 13q responsible for BAV and associated cardiac malformations, but the precise genes within these regions were not defined [9].

To date, only mutations in the *NOTCH1* gene, a signaling transmembrane receptor (2,556 amino acids) involved in multiple cell aspects of vascular development, have been associated with dominantly inherited BAV in a small number of families [10].

However, the rate of *NOTCH1* gene mutations in familial forms of BAV and their association with different clinical complications remains incomplete [11,12]. Interestingly, McBride et al. demonstrated that mutations in the gene *NOTCH1* are found in cases of left ventricular outflow tract (LVOT) malformations including aortic valve stenosis, coarctation of the aorta and hypoplastic left heart syndrome [13]. In particular, a much higher rate of bicuspid aortic valve (BAV) has been found in families with LVOT malformations compared to the general population, suggesting that BAV may be a *forme frusta* of the more serious LVOT malformations [13].

Recently, a missense mutation in transforming growth factor-beta receptor 2 gene (*TGFBR2)* was found in a patient with BAV and aortic aneurysm [14], but earlier studies found no mutation in either *TGFBR1* or *TGFBR2* in patients with familial and sporadic BAV disease [15,16].

Finally, *GATA5* was reported to have an essential role in cardiac morphogenesis and in aortic valve development [17,18], and targeted deletion of this gene in mice leads to partially penetrant BAV [19]. Accordingly, a very recent study investigated the relationship between *GATA5* gene variations and BAV disease identifying rare non-synonymous variations in the functionally important *GATA5* transcriptional activation domain [20]. Conversely from *NOTCH1* gene, the involvement of *GATA5* has not been described in other LVOT malformations.

However, further studies are needed to assess the involvement of *NOTCH1*, *GATA5* and *TGFBR1* and *TGFBR2* mutations in patients with BAV and its associated aortic complications.

The purpose of our study was to investigate the potential contribution of germline mutations in *NOTCH1, GATA5*, *TGFBR1* and *TGFBR2* genes in a cohort of Italian patients with familial BAV.

## Methods

### Study population

**Figure 1:**
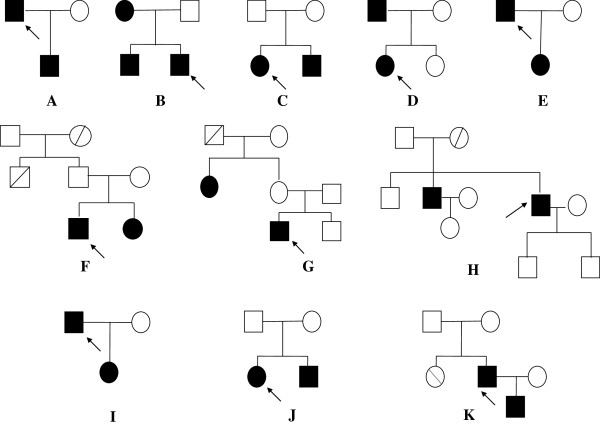
**Pedigrees of BAV families A-K.** The proband is indicated by arrow and affected status is indicated by filled symbol. Slanted bars represent deceased individuals.

A cohort of 11 unrelated Italian patients (8 males; age = 42 ± 19 years) with familial BAV were recruited for this study. Patients with sporadic BAV and/or with coexisting syndromes, such as Marfan syndrome, Turner syndrome and Di George syndrome or unknown syndromes were excluded from the study. Clinical evaluation consisted of medical history, physical examination, 2-dimensional echocardiography and a complete Magnetic Resonance Imaging (MRI) study. A detailed family history was obtained for each proband. Familial BAV was defined if two or more affected relatives had proven BAV diagnosed by echocardiography or cardiac magnetic resonance. Figure 1 shows the pedigree of BAV patients. Patient’s phenotype is reported in Table 1. Two of the probands (18%) had BAV with unknown morphology. Of the 9 probands with known BAV morphology, 7 (63%) had fusion of the right/left coronary commissure whereas 2 (18%) had a valve with anterior-posterior sinus without raphe.We also included a control cohort comprised of 100 ethnically unrelated individuals (60 male; age = 35 ± 16 years) without BAV as shown by echocardiography. This study was conducted with informed consent of every participant subject and was approved by the local Ethical Research Committee.

**Table 1:** Clinical and demographic characteristics of study population

**Proband**	**Sex**	**Age**	**Diagnosis**	**Phenotype**	**Other cardiac malformation**
**A**	M	40	AS and AAD	AP	/
**B**	M	25	AR and AAD	R-L	/
**C**	F	40	AR	unk	/
**D**	F	21	AR	R-L	CoA
**E**	M	37	BAV	R-L	/
**F**	M	74	AS and AAD	unk	/
**G**	M	17	AI and AAD	R-L	/
**H**	M	67	AR and AAD	R-L	/
**I**	M	38	AS	R-L	CoA
**J**	F	65	AS and AAD	AP	/
**K**	M	37	AR and AAD	R-L	/

AS: aortic stenosis; AAD: Ascending Aortic Dilation; AR: Aortic regurgitation; AI: aortic insufficiency; CoA: Aortic Coarctation; AP: anterior-posterior no raphe; R-L: right/left coronary commissure; unk: unknown.

### Mutational sequencing analysis

A mutational screening was performed by direct gene sequencing of all coding exons including adjacent intronic as well as 5^′^ and 3^′^ untranslated sequences of *NOTCH1*, *GATA5* and *TGFBR1*, *TGFBR2* genes. Genomic DNA was extracted from peripheral blood cells using the QIAGEN BioRobot® EZ1 System. Exons and the flanking intronic sequences were amplified by polymerase chain reaction (PCR) using specific primers as described previously for *NOTCH1* and *TGFBR1* and *TGFBR2*[11,21] and using Primer3 (v. 0.4.0) software (http://frodo.wi.mit.edu/primer3/) based on the cDNA sequences available in GenBank for *GATA5* screening.

Approximately, PCR amplifications were performed with a volume of 50 microl mixture containing 1.5 mM MgCl2, 10 mMdNTP, 50 ng genomic DNA, 20 micromol of each primer and 2.5 U Taq DNA polymerase. PCR product was used for PCR sequencing reaction with the CEQ DTCS Quick Start Kit. The sequencing reaction products were purified by precipitation with ethanol, re-suspended in a sample loading solution and analyzed with a CEQ 8800 capillary sequence (*Beckman Coulter, Germany*) according to the manufacturer’s protocol. Resulting sequences were analyzed using CEQ 8800 software packages and aligned against a reference sequence obtained from GenBank.

For prediction of the functional consequences of mutations on protein sequence, we used software programs available on the internet, namely PolyPhen-2(http://genetics.bwh.harvard.edu/pph/) SIFT (http://sift.jcvi.org/) and Mutation Taster (http://www.mutationtaster.org/*)*. PolyPhen-2 predicts the effect of an amino acid substitution on the structure and function of a protein, using sequence homology. The PolyPhen-2 score represents the probability that a substitution is damaging, so values nearer 1 are more confidently predicted to be deleterious (note that this is the opposite of SIFT).

SIFT uses sequence homology to predict whether an amino acid substitution will affect the protein function. SIFT score <0.05 indicates the amino acid substitution is damaging while scores ≥ 0.05 are predicted to be tolerant. Mutation Taster is a free, web-based application for rapid evaluation of the disease-causing potential of DNA sequence alterations. Mutation Taster integrates information from different biomedical databases and uses established analysis tools. Analyses comprise evolutionary conservation, splice-site changes, loss of protein features and changes that might affect the amount of mRNA.

Detected mutations in probands were confirmed by sequencing in the opposite direction from another two independent PCR products*.*

The presence of a novel mutation was assessed in 200 ethnically reference alleles derived from volunteers by PCR-based restriction fragment length polymorphism (PCR-RFLP) or by direct gene sequencing.

## Results

Clinical and demographic characteristics of each patient are summarized in Table 1. The group comprised 73% males, and coarctation of the aorta (CoA) as coexistent cardiovascular anomalies occurred in 18% of the cohort.

### Genetic analysis of *NOTCH1* gene

A mutational screening was performed by direct gene sequencing of all 34 coding exons including adjacent intronic as well as 5^′^ and 3^′^ untranslated sequences of *NOTCH1* gene. Seventeen separate *NOTCH1* sequence variants listed previously in public dbSNPs or in the literature were found in our patients, while five variants were novel. These included one synonymous variant (Exon 21, p.G1166G), two intronic (IVS11 + 63; IVS32-41) and two novel mutations (Exon 5,p.P284L; Exon 26, p.Y1619X). Details of the specific sequence variants and number of positive patients are summarized in Table 2.

**Table 2:** **Genetic variants within the human *NOTCH1 *gene identified in familial BAV patients**

**Genomic position (NG_007458.1)**	**Position *NOTCH1 *gene**	**cDNA position (NM_017617.3)**	**Protein position**	**Number of patients**	**rsSNP number (dbSNP)**
g.26979 T > C	Exon 3	c.312 T > C	p.N104N	6	rs4489420
g.31330 C > T	Exon 5	c.851C > T	**p.P284L**	1	**New**
g.31331 G > A	Exon 5	c.852 G > A	p.P284P	4	rs2229975
g.34650 T > C	IVS 9-43			3	rs4880099
g.33525A > G	IVS 9 + 10			2	rs11145767
g.33601 C > A/T	IVS 9 + 86			2	rs113341997
g.33617C > T	IVS 9 + 102			1	rs10781498
g.33620C > T	IVS 9 + 105			1	rs11574887
g.34772C > T	Exon 10	c.1635 C > T	p.D545D	1	rs11574889
g.35062A > G	IVS 11-9			3	rs3124603
g.35367 G > A	IVS 11 + 63			1	**New**
g.35591C > T	IVS 12 + 94			1	rs62579232
g.36347G > A	IVS 13 + 70			2	rs3812609
g.37307 T > C	Exon 14	c.2265 T > C	p.N755N	4	rs2229971
g.39978G > A	IVS 16-4			3	rs3125001
g.40058C > T	Exon 17	c.2664 C > T	p.H888H	1	rs61751548
g.40085C > T	Exon 17	c.2691 C > T	p.A897A	1	rs11574895
g.42820 C > T	Exon 21	c.3498 C > T	**p.G1166G**	1	**New**
g.45953 C > G	Exon 26	c.4856 C > G	**p.Y1619X**	1	**New**
g.47532C > T	Exon 27	c.5094 C > T	p.D1698D	3	rs10521
g.48831 T > C	IVS 30-43			2	rs3124594
g.49928C > T	IVS 30-12			1	rs11574908
g.51487 C > T	IVS 32-41			1	**New**

**Figure 2:**
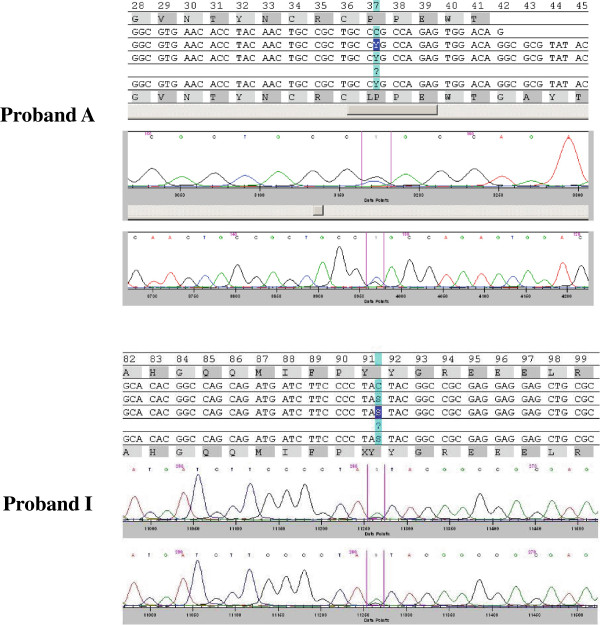
DNA sequencing electropherograms demonstrating the NOTCH1 heterozygous sequence mutations (Exon 5, p.P284L and Exon 26, p.Y1619X).

The novel C > T heterozygous substitution in exon 5, resulting in the substitution of a Proline with a Leucine (P284L) at position 284, was found in the sequencing electropherograms of a 40-year-old male patient (Proband A; Figure 2). The identified missense mutation was absent in 200 ethnically reference alleles derived from volunteers. PolyPhen-2 analysis predicted the p.P284L mutation as probably damaging with a score of 0.993. This result was confirmed by SIFT (score: 0.04).

The missense mutation (p.P284L) identified in our proband, tested by Mutation Taster, induced the loss of an important residue with putative significance for calcium binding to epidermal growth factor (EGF)-like 7 domain.

The proband, at the age of 39 years, presented severe calcified aortic valve and normal diameter of ascending aorta (39 mm). One year later, the patient underwent elective aortic valve and ascending aorta repair (48 mm) replacement. Family history and echocardiography analysis revealed the presence of BAV in his 7-year-old son. Genetic screening of the proband’s child confirmed the presence of p.P284L mutation in exon 5 of *NOTCH1* gene.

Moreover, we detected a novel heterozygous C-to-G transition, in exon 26, of nucleotide 4856 leading to a premature stop codon at position 1619 (p.Y1619X) instead of tyrosine in the sequencing electropherograms of a 38-year-old male patient (Proband I; Figure 2). The identified mutation was absent in 200 ethnically reference alleles derived from volunteers. At age 6 months the proband had surgery to correct the CoA, while at age 25 years he underwent elective aortic valve replacement. Family history and echocardiography analysis revealed the presence of BAV in his 5-year-old daughter. Genetic screening of the proband’s child confirmed the presence of p.Y1619X mutation in exon 26 of *NOTCH1* gene.

### Genetic analysis of *GATA5* gene

Sequencing of all 6 exons and splice signal sequences of *GATA5* in our cohort revealed five synonymous, one non-synonymous and three intronic variants. Details of the specific sequence variants and number of positive patients are summarized in Table 3. We identified one novel intronic variant (C > T; IVS 5–15) in one patient, but it was also present in our control group.

**Table 3:** **Genetic variants within the human *GATA5 *gene identified in familial BAV patients**

**Position *GATA5 *gene**	**HGVS names**	**Protein position**	**Number of patients**	**rsSNP number (dbSNP)**
Exon1	c.199 A > C	p.Thr67Pro	5	rs6142775
Exon 2	c.609 C > T	p.Asp203Asp	7	rs41305803
Exon 2	c.678 C > T	p.Leu226Leu	1	rs139428354
IVS 4-14	c.826-14 G > A		1	rs140408446
Exon 4	c.852 G > A	p.Lys284Lys	8	rs6587239
IVS 5-16	c.914-16 C > T		1	**New**
Exon 5	c.981 G > C	p.Ser327Ser	7	rs6061243
IVS 6-78	c.1039-78 C > G		5	rs113912772
Exon6	c.1128 A > G	p.Pro376Pro	11	rs6061550

NM_080473.4.

### Genetic analysis of *TGFBR1* and *TGFBR2* genes

*TGFBR1* and *TGFBR2* genes were analyzed by direct gene sequencing of all coding exons including adjacent intronic as well as 5^′^ and 3^′^ untranslated sequences. No pathogenetic mutation was identified; we found only known common genetic polymorphisms. Details of the specific sequence variants in *TGFBR1* and *TGFBR2* genes are summarized in Table 4.

**Table 4:** **Genetic variants within the human *TGFBR1 *and *TGFBR2 *genes identified in familial BAV patients**

**Gene**	**Position *TGFBR1 TGFBR2 *gene**	**HGVS names**	**Protein position**	**Number of patients**	**rsSNP number**
***TGFBR1***	Exon 1	c.68_76delCGGCGGCG	p.Ala23Ala	4	rs11466445
	IVS 7 + 24	c.1255 + 24 G > A		2	rs334354
***TGFBR2***	IVS 3 + 7	c.338 + 7 A > G		2	rs1155705
	IVS 5-4	c.455-4 T > A		3	rs11466512
	Exon 4	c.458delA	p.Lys153	8	rs79375991

*TGFBR1*: NM_004612.2 NG_007461.1.

*TGFBR2:* NM_001024847.2 NG_007490.1.

## Discussion

In our cohort of 11 familial patients with BAV two novel mutations were found in the *NOTCH1* gene in two unrelated families. The mutations segregated with the disease in these families, and they were not found in 200 chromosomes from ethnically matched controls.

On the contrary, analysis of *GATA5*, *TGFBR1* and *TGFBR2* genes identified only known genetic variants. Our findings confirm the important role of NOTCH1 as conserved intracellular regulator in the pathogenesis of congenital valve disease and in its complications. Indeed, the finding of two independent *de novo* mutations in this gene in a small number of affected subjects is compelling evidence of disease causation.

*NOTCH1* encodes for a transmembrane protein that activates a signaling pathway with a major role in cardiac embryogenesis, including aortic and pulmonary valve development as well as the development and maintenance of the aorta and other great vessels [22]. Murine model studies indicate that Notch signaling promotes the epithelial-to mesenchyme transition that gives rise to the primordial cardiac valve, which later is sculpted into mature valves [23]. Moreover, a recent study showed that heterozygous Notch1-null (Notch1(+/)(−) mice had greater than fivefold more aortic valve calcification than age- and sex-matched wild type littermates [24].

Indeed, *in vitro* studies showed that Notch1 signaling repressed valvular Bmp2 expression, and de-repression of Bmp2 was involved in calcification induced by Notch1 inhibition. Thus, Notch1 signaling appears to prevent aortic valve calcification in part by repressing Bmp2 expression within the valve [24].

Defective NOTCH signaling might also contribute to aortic valve calcification by repressing the activity of the hairy-related family of transcription factors (Hrt), and thereby also repressing the Runx2/Cbfa1 pathway, a central transcriptional regulator of osteoblast cell fate [24,25].

In addition, there is increasing evidence of interaction between the Notch1 pathway and transforming growth factor beta (TGFβ) [26]. This is particularly important in light of evidence showing that TGFβ plays an important role in vascular remodeling and alterations in TGFβ signaling activity might enhance matrix degradation [27,28].

In 2005, the discovery of a nonsense (p.R1108X) and a frameshift mutation (p.H1505del) in two families represented the first demonstration of *NOTCH1* germline mutations as a cause of BAV and severe valve calcification in non-syndromic autosomal-dominant human pedigrees [10].

Subsequently, two novel mutations (p.T596M and p.P1797H) located in highly conserved regions of the protein were identified in patients with sporadic BAV, providing evidence that mutations in *NOTCH1* do not only play a role in familial BAV, but can also be observed in approximately 4% of sporadic cases [11]. Furthermore, a targeted mutational analysis of *NOTCH1* demonstrated the presence of two novel mutations (p.A1343V and p.P1390T) among patients with BAV with sporadic and concomitant thoracic aortic aneurysms, providing evidence that mutations in *NOTCH1* may also confer susceptibility to aortic aneurysm formation [12].

In a very recent paper, Kent *et al*. suggested a NOTCH1-dependent mechanism that produces stenotic, insufficient and/or calcified aortic valves with a rare aneurysm, and a NOTCH1-independent mechanism that produces highly penetrant ascending aortic aneurysm (AscAA) in the presence of a non-calcified and often normally functioning BAV [29]. Our data appear to agree with and fit well into the proposal made in this article by Kent *et al*., since we found two novel mutations in these patients with valve malformation, calcification, and dysfunction as predominant phenotype caused by altered NOTCH1 signaling.

Conversely, we did not identify any pathogenetic mutations in *GATA5*. The non-synonymous variant Thr67Pro located in the transcriptional activator domain encoded by exon 1, detected with a frequency of 45% in our population, is a common genetic polymorphism previously described [20,30].

Regarding to *TGFBR1* and *TGFBR2* genes, in contrast with a recent paper showing a missense mutation in *TGFBR2* gene in a patient with BAV and aortic aneurysm [14], our findings support the studies suggesting that mutations in these genes are rarely identified in patients with familial BAV [15,16].

## Conclusion

Although the small number of patients (11) might be an important study limitation, we have identified two novel *NOTCH1* mutations in two Italian families with BAV. Our findings confirm the critical role of *NOCTH1* in the development of aortic valve and aortic complications. Genetic screening for *NOTCH1* mutation may be considered for diagnosis and family screening as part of the standard management of patients with family history of BAV. However, the etiology of this common valve disease is largely unknown. Future studies based on new genetic screening strategies, the so-called next-generation sequencing, are needed in order to unravel the still largely unknown genetics of BAV.

Abbreviations: BAV = Bicuspid Aortic Valve, *TGFBR1 =* transforming growth factor-beta receptorI gene; *TGFBR2 =* transforming growth factor-beta receptorII gene; PCR-RFLP = PCR-based restriction fragment length polymorphism; MAF = minor allele frequency; CoA = coarctation of the aorta; TGFβ = transforming growth factor beta; MRI = Magnetic Resonance Imaging; AscAA = ascending aortic aneurysm; LVOT = left ventricular outflow tract.

## Competing interests

The authors declare that they have no competing interests.

## Authors’ contributions

IF participated in the design of the study, carried out the molecular genetic studies, performed the statistical analysis and drafted the manuscript. MGA conceived the study, participated in its design and coordination and critically revised the manuscript. PP carried out the molecular genetic studies. MM, AAL and PF participated in design of the study and in the enrollment of the patients. NB and CV performed the genetic analysis and critically revised the manuscript. All authors read and approved the final manuscript.

## Pre-publication history

The pre-publication history for this paper can be accessed here:

http://www.biomedcentral.com/1471-2350/14/44/prepub
